# A resveratrol oligomer, hopeaphenol suppresses virulence activity of *Pectobacterium atrosepticum via* the modulation of the master regulator, FlhDC

**DOI:** 10.3389/fmicb.2022.999522

**Published:** 2022-10-28

**Authors:** Ji Eun Kang, Sungmin Hwang, Nayeon Yoo, Beom Seok Kim, Eui-Hwan Chung

**Affiliations:** ^1^Institute of Life Science and Natural Resources, Korea University, Seoul, South Korea; ^2^Clean Energy Research Center, Korea Institute of Science and Technology (KIST), Seoul, South Korea; ^3^Department of Plant Biotechnology, Graduate School, Korea University, Seoul, South Korea; ^4^Division of Biotechnology, College of Life Sciences and Biotechnology, Korea University, Seoul, South Korea

**Keywords:** anti-virulence agent, bacterial motility, *flhDC*, hopeaphenol, *Pectobacterium*

## Abstract

*Pectobacterium atrosepticum* (*P. atrosepticum*: *Pba*) which causes potato soft rot and blackleg is a notorious plant pathogen worldwide. Discovery of new types of antimicrobial chemicals that target specifically to virulence factors such as bacterial motility and extracellular enzymes is required for protecting crops from pathogenic infection. A transcriptomic analysis of *Pba* upon hopeaphenol treatment revealed that bacterial motility-related gene expression, including a master regulator *flhDC* genes, was significantly influenced by hopeaphenol. We further generated a double knock-out mutant of *flhDC* genes by CRISPR/Cas9 system and confirmed phenotypic changes in bacterial motility, transcription of extracellular enzymes, and disease development consistent with the result of wild-type treated with hopeaphenol. The hopeaphenol-treated *Pba* strains, wild-type, double mutant, and complemented strain were unable to secrete the enzymes *in vitro*, while Δ*flhDC* double mutant strain reduced the secretion. Thus, our study supports that FlhDC is essential for the virulence of *Pba*, and proposes that hopeaphenol modulates FlhDC-dependent virulence pathways, suggesting a potential of hopeaphenol as an anti-virulence agent to manage potato soft rot and blackleg diseases.

## Introduction

*Pectobacterium atrosepticum* belongs to Enterobacteriaceae family and causes soft rot and blackleg development on potato plants in the field (Bain et al., 1990). Soft rot Pectobacteriaceae including *Pectobacterium* spp. and *Dickeya* spp. was ranked in the top 10 plant pathogenic bacteria in 2010, as the world has been suffering from potato soft rot and blackleg, leading to major economic losses due to reduced yield and quality (Mansfield et al., 2012; Kumvinit and Akarapisan, 2019). For successive cultivation of potatoes, seed potatoes are prepared clonally, and the seeds are tightly controlled to prevent bacterial infection (Dupuis et al., 2021). *Pba* colonizes plant vessels without visible symptoms followed by, upon successful infection, emerging maceration of infected plant tissues with diverse lytic extracellular enzymes and spreading to the whole potato tuber. Macerated tissue turns to black color frequently with an odd smell in the presence of air (Czajkowski et al., 2011). Control strategies against *Pba*-induced disease have been studied intensively, but until now, efficient commercial control agents for soft rot and blackleg diseases are very limited (Wolf et al., 2021). The traditional method which only focuses on seed certification is widely adopted in agriculture, and physical and chemical treatments such as hot water, hot dry air, steam, UV, or antibiotics are deployed to seed potatoes to reduce *Pectobacterium* population in latently infected potatoes (Charkowski, 2015). However, these control methods to eradicate such pathogens have a limit due to the infection emerging post-harvest (Pérombelon, 1992; Czajkowski et al., 2011). The application of biocontrol agents emerges as an alternative and complementary to the traditional method. Crépin et al. (2012) reported that soil bacterium *Rhodococcus erythropolis* (*R. erythropolis*) degrades the quorum sensing molecule of *Pba* disrupting bacterial communication and consequently reducing blackleg disease with no alteration of growth and transcriptional changes in avirulent *Pba* strain, while *R. erythropolis* affects QS-controlled virulence phenotypes in the virulent *Pba* strain. This indicates that *R. erythropolis* is a promising biological control agent that dampens the activity of quorum sensing molecules of *Pba* (Kwasiborski et al., 2015).

The production of extracellular enzymes including pectate lyase (Pel), polygalacturonase (Peh), protease (Prt) and cellulase (Cel) is major virulence determinant of necrotrophic pathogen *Pba* (Smadja et al., 2004). These enzymes degrade components of the plant cell wall, resulting in the maceration of plant tissue which is a representative soft-rot disease symptom. The quorum sensing (QS) system controls the production of extracellular enzymes along with an assortment of transcriptional and post-transcriptional regulators (Chatterjee et al., 2009). In *P. carotovorum,* a master regulator (FlhDC) is required for normal exoenzyme production (Bowden et al., 2013). The FlhDC complex facilitates extracellular enzymes production by relieving the repressor HexA during exoenzyme biosynthesis (Cui et al., 2008). FlhDC regulates the transcription level of class II flagellar regulon genes (i.e., encoding for hook and basal body of flagellum) positively as well. The class II regulon genes such as *fliA* subsequently activates the transcription of the class III regulon for flagella filament formation, chemotaxis machinery, and motor protein complex (Bowden et al., 2013).

Bacterial motility by flagella facilitates bacterial movement to favorable environments or escape from detrimental conditions for successful competition with other organisms (Hossain et al., 2005). Flagella-driven motility also mainly contributes to pathogenic infection and disease development (Jahn et al., 2008). The non-motile mutants of *P. carotovorum* subsp. *carotovorum* (*Pcc*) compromised soft rot disease in Chinese cabbage (Hossain et al., 2005). Mop (motility and pathogenicity) proteins of *Pba* are presumably involved in flagella production and export of flagellar proteins. *Pba mop* mutant which is a non-motile strain was demonstrated to be reduced in both virulence activity without bacterial growth defect and exoenzyme production (Mulholland et al., 1993).

The identification of natural compounds to control plant bacterial diseases is fundamental for crop protection due to their diverse structures and pharmacophores inspiring the design of new drugs (Sekurova et al., 2019; Raymaekers et al., 2020). Natural QS inhibitors, piericidin A and glucopiericidin A isolated from *Streptomyces xanthocidicus* inhibit the transcription of QS controlled virulence genes and reduce *Erwinia* soft rot disease in potato plants (Kang et al., 2016). The active compound piericidin A was firstly isolated as an insecticidal agent acting as a NADH–ubiquinone oxidoreductase inhibitor for disrupting mitochondrial respiratory chain (Tamura et al., 1963; Liu et al., 2012). Since then, various biological activities in view of insecticidal, antimicrobial, anti-tumor activities, cytotoxicity, and type III secretion system (T3SS) inhibition have been studied intensively (Tamura et al., 1963; Morgan et al., 2017; Li et al., 2021; Azad et al., 2022).

Hopeaphenol displayed anti-virulence activity against hemi-biotrophic bacterial pathogen *Pseudomonas syringae* pv. *tomato* DC3000 (*Pst* DC3000) by inhibiting T3SS and bacterial motility (Kang et al., 2020, 2022). In this study, we further examined anti-virulence activity of hopeaphenol on the necrotrophic bacterial pathogen *Pba.* We analyzed whole transcriptome to identify how *Pba* responds to hopeaphenol in the transcription level. We further investigated the role of a master regulator FlhDC responsible for the bacterial pathogen motility and extracellular enzyme production regarding the virulence activity of *Pba* by employing CRISPR-Cas9-mediated mutant strain generation. Overall, our study is the first to report hopeaphenol as a biological control agent for the necrotrophic bacterial pathogen *Pba* by modulating bacterial motility, secretion and transcription of extracellular enzymes leading to disease resistance in the host plant.

## Materials and methods

### Bacterial RNA extraction and transcriptomic analysis

*Pba* was overnight cultured in Luria-Bertani (LB) medium, resuspended in Pel minimal medium (0.1% yeast extract, 0.1% (NH_4_)_2_SO_4_, 1 mM of MgSO_4_, 0.5% glycerol and 0.5% polygalacturonic acid in 50 mM of phosphate buffer, pH 7.0) with 100 μM of hopeaphenol or 0.2% acetone as a mock control to an optical density (OD) at 600 nm of 0.8, and incubated for 18 h at 18°C at 180 rpm. The NucleoSpin® RNA kit (Macherey-Nagel) was used to extract total RNA from the cell culture. RNA concentration and integrity were determined by using an Agilent Technologies 2,100 Bioanalyzer. Qualified samples based on the RNA integrity number (higher than 9.0) were further proceeded to construct sequencing libraries by a TruSeq Total RNA (NEB Microbe) kit. Paired-end sequencing of the constructed cDNA libraries was performed under the Illumina Hiseq X Ten platform by Macrogen, Inc. (Seoul, Korea). About 20 million raw sequencing reads were generated, and FastQC v.0.11.7^1^ was applied to remove the adapter, poor-quality and short reads (< 10 bp). The preprocessed reads were aligned to the reference genome of *Pba* SCRI1043^2^ by Bowtie2 (Langmead and Salzberg, 2012), followed by sorting and indexing by Samtools (Li et al., 2009). HTSeq was used to count the number of reads mapped to each transcript (Anders et al., 2015). The read counts were normalized and differentially expressed genes (DEGs) were identified by using DESeq2 (Love et al., 2014). Genes with a log_2_ fold change greater than 1 and a false discovery rate (FDR) determined by Benjamini–Hochberg (BH) correction for multiple hypothesis testing of less than 0.05 were considered as DEGs. Functional enrichment was performed by the hypergeometric test from the eggNOG 4.5 orthology database (Huerta-Cepas et al., 2016). Pathway enrichment was analyzed by Pathview Web (Luo et al., 2017). All RNA-seq data were submitted to NCBI and NCBI assigned accession number is GSE196675.

### Bacterial mutant strain

CRISPR-Cas9-based genome editing was used to construct Δ*flhDC* strain as described in the previous study (Wang et al., 2020). Briefly, 20 bp of spacer oligonucleotides in *flhDC* gene of *Pba* SCRI1043 were designed using sgRNAcas9 software (Supplementary Table S1; Xie et al., 2014). Phosphorylated oligonucleotides were inserted into pSGAb-km by the Golden Gate assembly, which generated pSGAb-km-*flhDC*. pCasAb-apr was transferred into wild-type *Pba* SCRI1043 electrocompetent cell. After selection with suitable antibiotics on LB medium, cell harboring pCasAb-apr was transformed with 200 ng of pSGAb-km-*flhDC* and 100 μM of 80 nt ssDNA donor DNA for *flhDC* gene using electroporation (Supplementary Table S1). The successfully transformed cell was confirmed by colony PCR with *flhDC* primer (Supplementary Table S1) and was cured on LB medium containing 5% sucrose *via sacB*-counter selection to obtain Δ*flhDC* strain. To construct complemented strain, *flhDC* genes were inserted into pBBR1MCS2 containing constitutive promoter (Addgene, #85168) and the plasmid was transformed into Δ*flhDC* mutant to generate Δ*flhDC*(p*flhDC*) strain.

### Bacterial growth

Wild type, Δ*flhDC* mutant, and complemented strain were grown in LB medium at 28°C for 16 h in shaking incubator at 200 rpm. Overnight cultured cells were adjusted to OD at 600 nm of 0.1. 200 μL of cell suspension was supplemented with 100 μM of hopeaphenol and dispended into 96-well plate followed by incubation at 28°C with 200 rpm for 16 h. The optical density was measured every 2 h by exponential phase and every 4 h after exponential phase. All procedures were followed by the methods described in Kang et al. (2020). The experiment was conducted three times with three technical replicates.

### Chemical compound

Hopeaphenol was isolated from a root extract of grapevine as described in Kang et al. (2020). Briefly, the roots were extracted with methanol, followed by Diaion HP-20 column chromatography with a methanol/water gradient (0–100%). By using high-performance liquid chromatography, the fraction obtained from the column chromatography was eluted with a linear gradient solvent system (10–80% aqueous methanol for 30 min followed by isocratic elution with 80% aqueous methanol for 10 min). The elution corresponding to hopeaphenol was collected for the following experiments.

### Bacterial motility assay

Bacterial motility assay was conducted as in the previous study (Chatterjee et al., 2009). *Pba* strains cultured in LB broth overnight were diluted to a final OD of 2.0 at 600 nm. 2 μL of diluted bacterial suspension was loaded onto motility medium (5 g of NaCl, 10 g of tryptone, and 3 g of agar per L). Hopeaphenol was supplemented at the final concentration of 100 μM into the medium. Bacterial migration was observed at 28°C for 48 h, and the net distance that bacteria moved was recorded with three replicates.

### Extracellular enzyme assay

Pel and Peh assays were performed according to the previously published procedure with some modification (Cui et al., 2008). Briefly, *Pba* strains were cultured in a minimal medium (2 g of KH_2_PO_4_, 7 g of K_2_HPO_4_, 0.1 g of MgSO_4_·7H_2_O, 1 g of (NH_4_)_2_SO_4_ and 5 g of sucrose per L) supplemented with 100 μM of hopeaphenol at 28°C for 24 h. 15 μL of bacterial culture supernatants was loaded onto Pel (1% polygalacturonic acid, 1% yeast extract, 0.38 μM of CaCl_2_, 100 mM of Tris–HCl, 0.8% agarose and 0.2% sodium azide, pH 8.5) and Peh (1% polygalaturonic acid, 1% yeast extract, 2.2 mM of EDTA, 110 mM sodium acetate, 0.8% agarose and 0.2% sodium azide, pH 5.5) medium and incubated at 28°C for 36 h. The Pel and Peh assay plates were developed with 4 N of HCl. Enzymatic activities of Pel and Peh were determined by a diameter measurement. The experiment was conducted three times with three technical replicates.

### Quantitative reverse transcriptional PCR

RNA extraction and cDNA synthesis were followed as described by Kang et al. (2016) with minor modification. Bacterial cells from overnight incubation in LB medium were resuspended in Pel minimal medium to OD_600_ of 0.8. *Pba* strains were incubated at 18°C for 18 h supplemented with 100 μM of hopeaphenol or 0.2% acetone as control. Bacterial total RNA was extracted, and cDNA was synthesized from 500 ng of total RNA. The same quantity of cDNA from each treatment was used as a template for qRT-PCR with primers listed in Supplementary Table S1. PCR amplification was conducted using Bio-rad CFX-96 as described in the manufacturer’s protocol. *gyrA* of *Pba* was used for internal control. The normalized gene expression was calculated as the ratio = 2^ΔCt(target gene)^/2^ΔCt(internal control gene)^. The experiment was performed three times with three replicates independently.

### Pathology assay

Potato tubers (*Solanum tuberosum* L.) were used to examine the effect of hopeaphenol and the mutation of *flhDC* genes on disease development by *Pba* strains used in this study. Potatoes were surface sterilized with 1% of sodium hypochlorite solution for 1 min and rinsed with 70% of ethanol once followed by washing with sterile water. After air-drying in the clean bench, yellow tip was inserted in the potato tubers with 20 mm depth. Bacterial cells from each *Pba* strains grown overnight were diluted with sterile water to optical density of 1.0 at 600 nm. Bacterial inoculum (10 μL) was applied to the wound site sealed with Vaseline. For the treatment of hopeaphenol, bacterial suspension supplemented with 100 μM of hopeaphenol was inoculated into the wound site. The potato tubers were incubated at 28°C for 3 days under high-humid condition. Disease progress and symptom development such as soft rot and blackleg were observed every day. After 3 days post-inoculation, macerated tissue around the wound sites was scraped off, weighed and diluted in sterile water. The bacterial population in the tissue was evaluated on LB agar plate at 28°C for 24 h. The bacterial population was determined by counting colonies on the plate.

## Results

### Hopeaphenol modulates transcriptional dynamics in *Pba* SCRI1043

**Figure 1:**
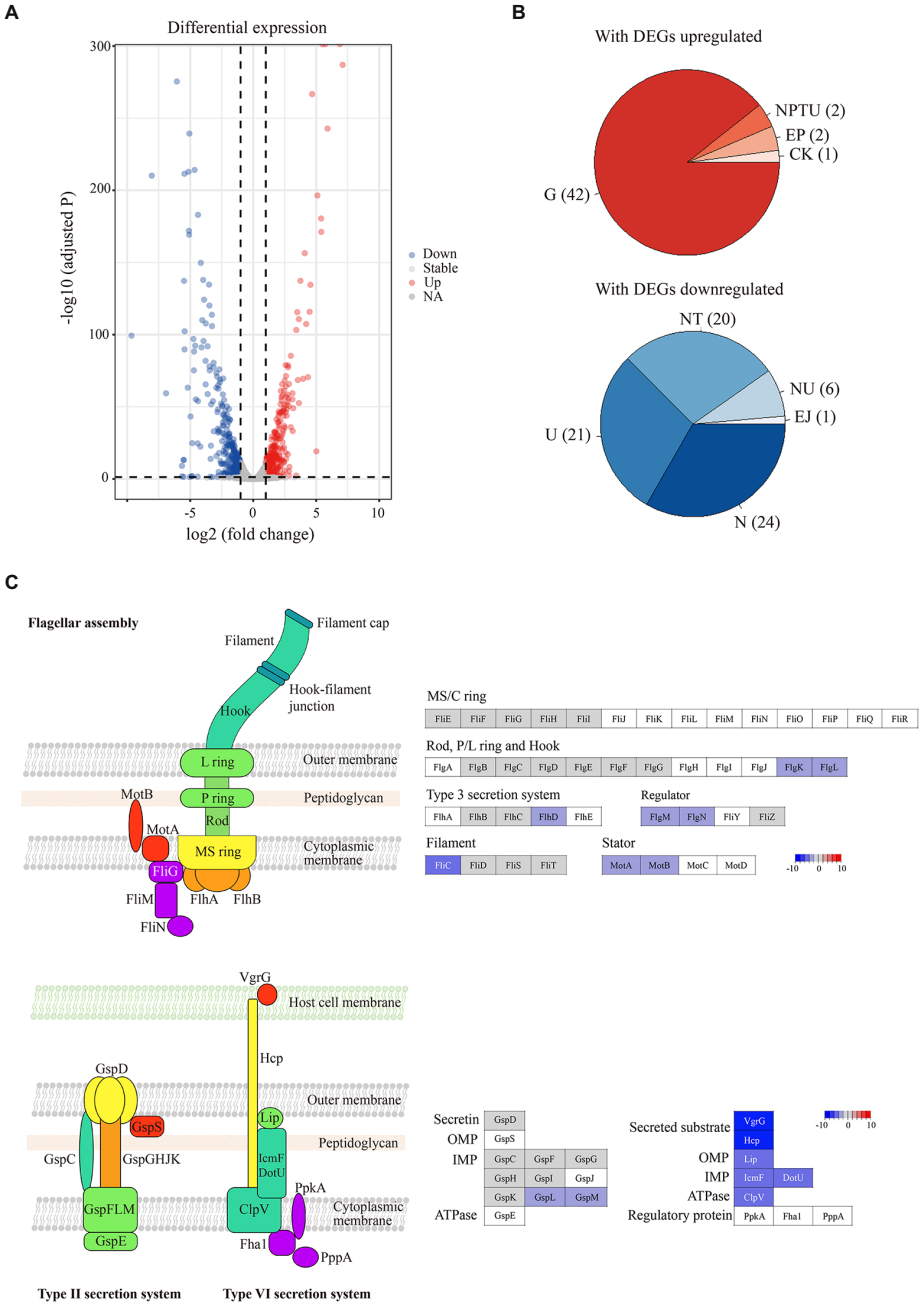
Hopeaphenol induces transcriptional changes in *Pba*. **(A)** Volcano plot to demonstrate differentially expressed genes in *Pba* by hopeaphenol-treatment. A total of 689 genes that are differentially expressed (log_2_ fold change >1 and padj <0.05, designated as DEGs) by hopeaphenol in *Pba* SCRI1043 are indicated in color. Red dots, genes upregulated by the comparison of conditions (hopeaphenol-treated/non-treated); blue, genes downregulated; grey, genes that expressed not significantly. **(B)** Clusters of orthologous groups (COGs) enrichment analysis with DEGs that up or downregulated. The statistically significant categories (*p* < 0.05) were shaded by the number of DEGs. COG category: C, energy production and conversion; E, amino acid transport and metabolism; G, carbohydrate transport and metabolism; J, translation, ribosomal structure and biogenesis; K, transcription; N, cell motility; P, inorganic ion transport and metabolism; T, signal transduction mechanisms; U, intracellular trafficking, secretion, and vesicular transport. **(C)** Schematic architecture of flagella and secretion systems with the gene expression of the corresponding components. The color key (from −10 to 10 scale) indicates the log_2_ gene expression ratio in hopeaphenol: non-treated and genes that are not DEGs colored in white.

To investigate the genome-wide gene expression profile of *Pba* SCRI 1043 upon hopeaphenol treatment, transcriptome analysis was conducted by using RNA-seq. Ten million reads were generated from all samples and the range of mapping rate to the reference genome was 96–99%. Compared to the non-treated control group, a total of 689 genes (15% of whole genes encoded in *Pba* SCRI 1043) were found to be differentially expressed in the hopeaphenol-treated condition (Supplementary Table S2). Particularly, 336 and 353 genes were up- and down-regulated in the presence of hopeaphenol, respectively (Figures 1A,B; Supplementary Table S2).

We then performed a functional enrichment analysis with the differentially expressed genes (DEGs). The G category (carbohydrate transport and metabolism) demonstrated the majority of the upregulated DEGs. Among the downregulated DEGs, N (cell motility), T (signal transduction mechanisms), and U (intracellular trafficking, secretion, and vesicular transport) categories were affected by hopeaphenol mainly (Supplementary Table S3). All DEGs were further clustered into several groups by the pathway enrichment assigned with value of *p* below 0.05. Most up-regulated DEGs by hopeaphenol in *Pba* SCRI1043 were related to 13 metabolic pathways including degradation of fatty acid, geraniol, amino acids, benzoate, and caprolactam, and metabolism of starch, sucrose, *β-*alanine, butanoate, pyruvate, pentose, and glucoronate (Supplementary Table S4). Among the 353 down-regulated DEGs were grouped into 6 categories including flagellar assembly, chemotaxis, secretion system, two-component system, citrate cycle (TCA cycle), and thiamine metabolism (Supplementary Table S5). Thus, we noted that the pathway involved in bacterial motility such as flagellar assembly and chemotaxis was the top candidate category influenced by hopeaphenol-treatment and 18 genes involved in flagellar machinery apparatus including basal body, hook and filament were regulated by the treatment of hopeaphenol (Figure 1C and Supplementary Table S5). Interestingly, hopeaphenol affected the expression of master regulator genes, *flhC* and *flhD,* of which the product regulates the expression of flagellar genes in *Pba* (Bowden et al., 2013). We also confirmed the expression of *flhC* and *flhD* in *Pba* by qRT-PCR analysis with hopeaphenol-treatment (Supplementary Figure S1). Together, RNA-seq results represented not only the comprehensive transcriptional changes in *Pba* SCRI1043 but reduced expression of cell motility-related genes, maybe inhibiting the flagella synthesis in the presence of hopeaphenol (Supplementary Data sheet 2).

### The growth of *Pba* is independent of the master regulator genes

**Figure 2:**
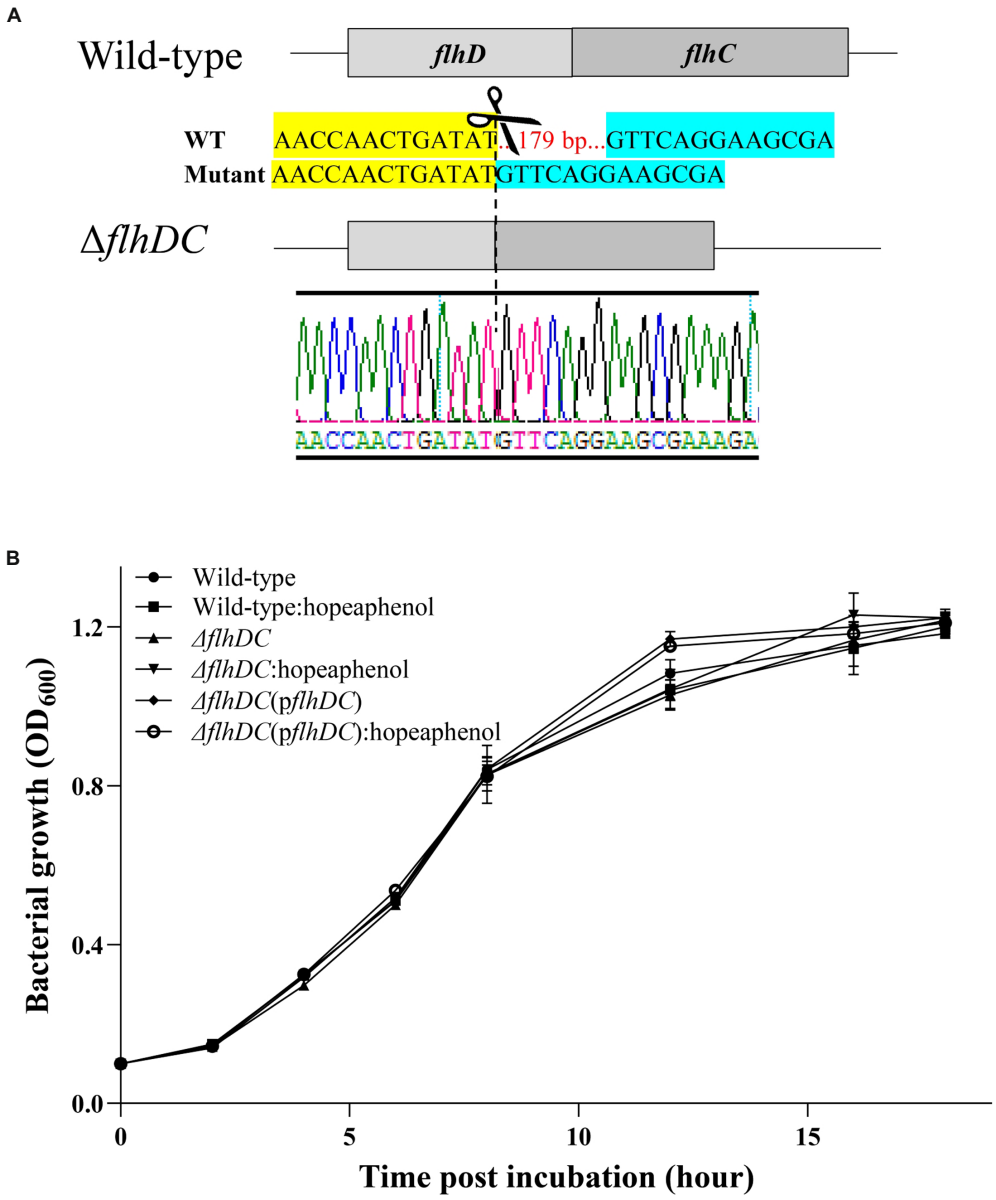
*Pba*-growth is independent of the master regulator *flhDC* genes. **(A)** A schematic illustration of CRISPR/Cas9-mediated editing of *flhDC* genes. 179 nt deletion within *flhDC* operon generated a clean double knockout mutant strain of *Pba* (Δ*flhDC*). **(B)** Bacterial growth *in vitro*. The growth of each strain (wild-type, Δ*flhDC,* Δ*flhDC*(p*flhDC*)) supplemented with 100 μM of hopeaphenol was measured by detecting optical density at 600 nm. The wild-type *Pba* strain without hopeaphenol was used to demonstrate the normal growth control. Each time point represents the average value of OD_600 nm_ of triplicate with error bars representing the standard error.

In *Pectobacterium*, flagellum motility and production of exoenzyme are important virulence factors, and their transcription is regulated by FlhDC complex (Cui et al., 2008; Mole et al., 2010). Considering the transcriptome analysis and qRT-PCR results mentioned above, we hypothesized that hopeaphenol could influence the expression of the master regulator gene (*flhDC*), leading to the change in virulence function. Thus, we generated a clean double knock-out mutant *Pba* (Δ*flhDC*) by employing CRISPR/Cas9 system (Figure 2A). As *flhC* and *flhD* work as an operon, double knock-out strain of *flhDC* was obtained by using one 20 bp-spacer introduced sgRNA and Cas9. The coding regions of *flhD*_194-351_ and *flhC*_1-58_ were successfully deleted ((Δ*flhDC*), Figure 2A). With the newly generated mutant strain, we also produced a plasmid-based complemented strain (Δ*flhDC*(p*flhDC*)). Transcript of *flhDC* was not detected in Δ*flhDC* which was recovered in complemented strain (Δ*flhDC*(p*flhDC*)) (Supplementary Figure S2). First, we monitored bacterial growth of *Pba* wild-type, mutant strain (Δ*flhDC*), and complemented strain of *flhDC* (Δ*flhDC*(p*flhDC*)) with or without 100 μM of hopeaphenol to address whether hopeaphenol possesses anti-bacterial activity. As in Figure 2B, all tested strains with or without hopeaphenol-treatment grew similarly. Therefore, our results indicate that hopeaphenol does not possess direct antibacterial activity to *Pba* and the master regulator genes may be dispensable for bacterial growth due to no change in growth in the presence/absence of hopeaphenol, leading us to investigate the role of hopeaphenol further in the virulence function of *Pba.*

### Hopeaphenol affects the bacterial motility of *Pba in vitro*

**Figure 3:**
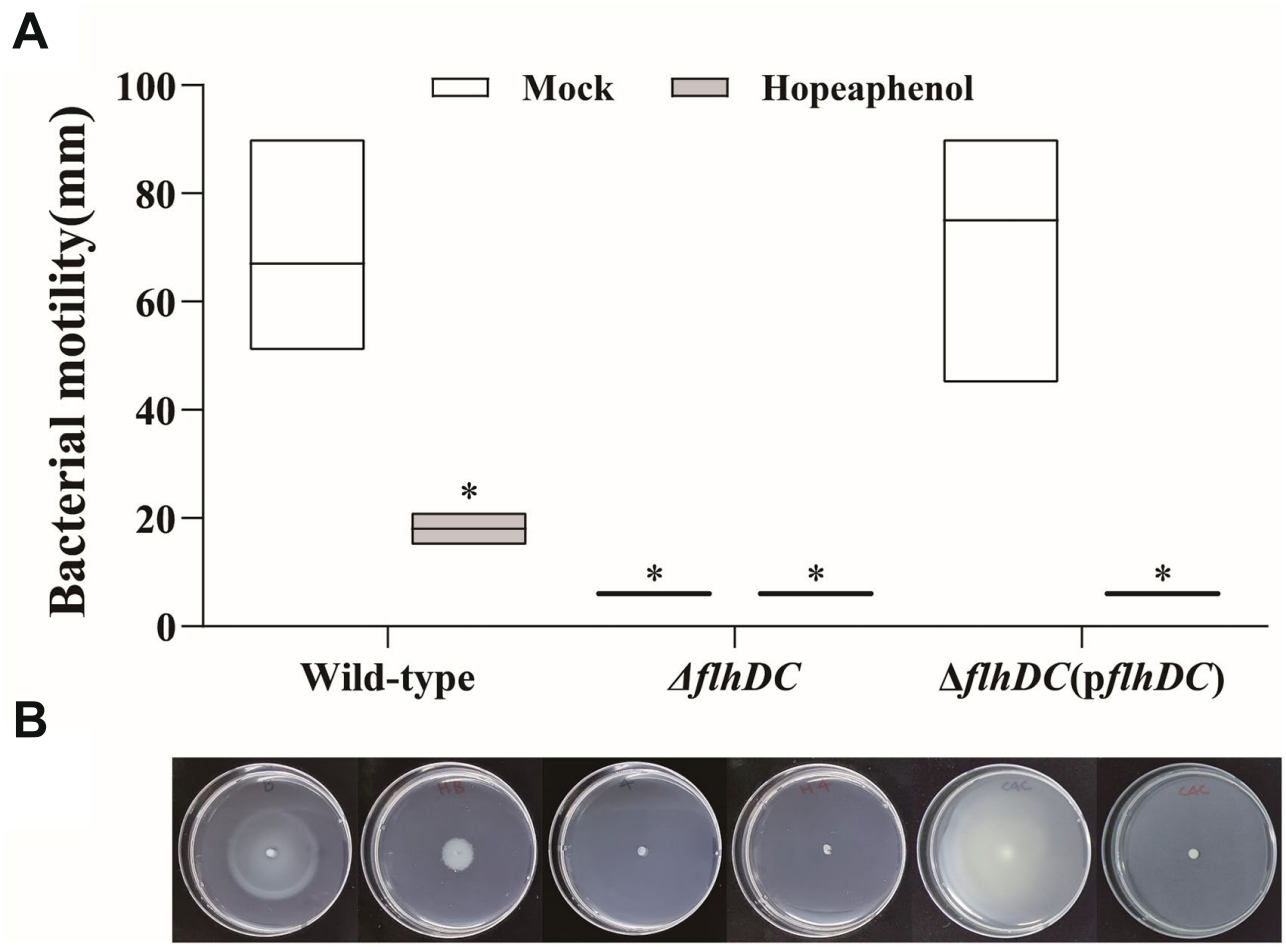
Bacterial motility of *Pba* is compromised in the wild-type strain with exogenous application of hopeaphenol and loss-of-function mutant of master regulator genes. **(A)** The motility phenotype of *Pba* strains with or without hopeaphenol. The bacterial motility was determined after 48 h of incubation by measuring the total moving distance (*n* = 3). The moving distance was measured with the diameter of the bacterial colony. The box-splitting horizontal line represents the mean, and the upper and the lower line display the highest and the lowest value. Asterisks demonstrate statistically significant differences based on the least significant difference (LSD) test at *p* < 0.05. **(B)** The image of bacterial motility. The representative pictures from three independent experiments were presented matched to the graph in **(A)**.

To further verify the role of hopeaphenol regarding *Pba* virulence activity *via* master regulator complex function, swimming motility phenotype was tested with *Pba* wild-type, double mutant strain (Δ*flhDC*), and complemented strain (Δ*flhDC*(p*flhDC*)) *in vitro*. Deletion of *flhC* and *flhD* in *Pba* compromised swimming ability as similarly in hopeaphenol-treated wild-type strain, proposing that hopeaphenol could suppress the motility of *Pba* by inhibiting the function of master regulator FlhDC (Figure 3). Complementation of the mutant strain with *flhDC* (Δ*flhDC*(p*flhDC*)) restored the motility as wild-type, and hopeaphenol-treatment retained the ability to inhibit bacterial motility in complemented strain (Figure 3). Thus, we concluded that hopeaphenol can modulate bacterial motility by the inhibition of master regulator FlhDC function.

### Hopeaphenol abrogates transcription and secretion of *Pba* exoenzymes

We then focused on another key virulence determinant in *Pba*, extracellular enzyme production including pectate lyase (Pel) and polygalacturonase (Peh) responsible for cell wall degradation and soft rot disease developments. The enzyme secretion of *Pba* strains in response to hopeaphenol was assessed in semi-quantitative assay for Pel and Peh activities. Deletion of *flhDC* genes reduced the activities of Pel and Peh by 26 and 49%, respectively (Table 1). Complemented strain (Δ*flhDC*(p*flhDC*)) restored the extracellular enzyme secretion to the level of wild-type (Table 1). However, hopeaphenol-treatment dramatically inhibited Pel and Peh secretion with no activity in all tested strains (Table 1).

**Table 1:** The activity of extracellular enzymes of *Pba* strains with hopeaphenol.

	Wild-type	Wild-type: hopeaphenol	Δ*flhDC*	Δ*flhDC*: hopeaphenol	Δ*flhDC*(p*flhDC*)	Δ*flhDC*(p*flhDC*): hopeaphenol
Pel^a^	1.94 ± 0.14^c^ a	ND^d^	1.43 ± 0.07 b	ND	2.09 ± 0.02 a	ND
Peh^b^	1.73 ± 0.12 a	ND	0.88 ± 0.02 b	ND	1.85 ± 0.03 a	ND

a Pel, pectate lyase.

b Peh, polygalacturonase.

^a,b^ Enzyme activity was expressed by a diameter of clear zones (cm) on pel and peh agarose plate.

c Means ± standard deviation of three replicates followed by different letters indicating significantly different based on the least significant difference test at *p* < 0.05.

d ND, not detected.

**Figure 4:**
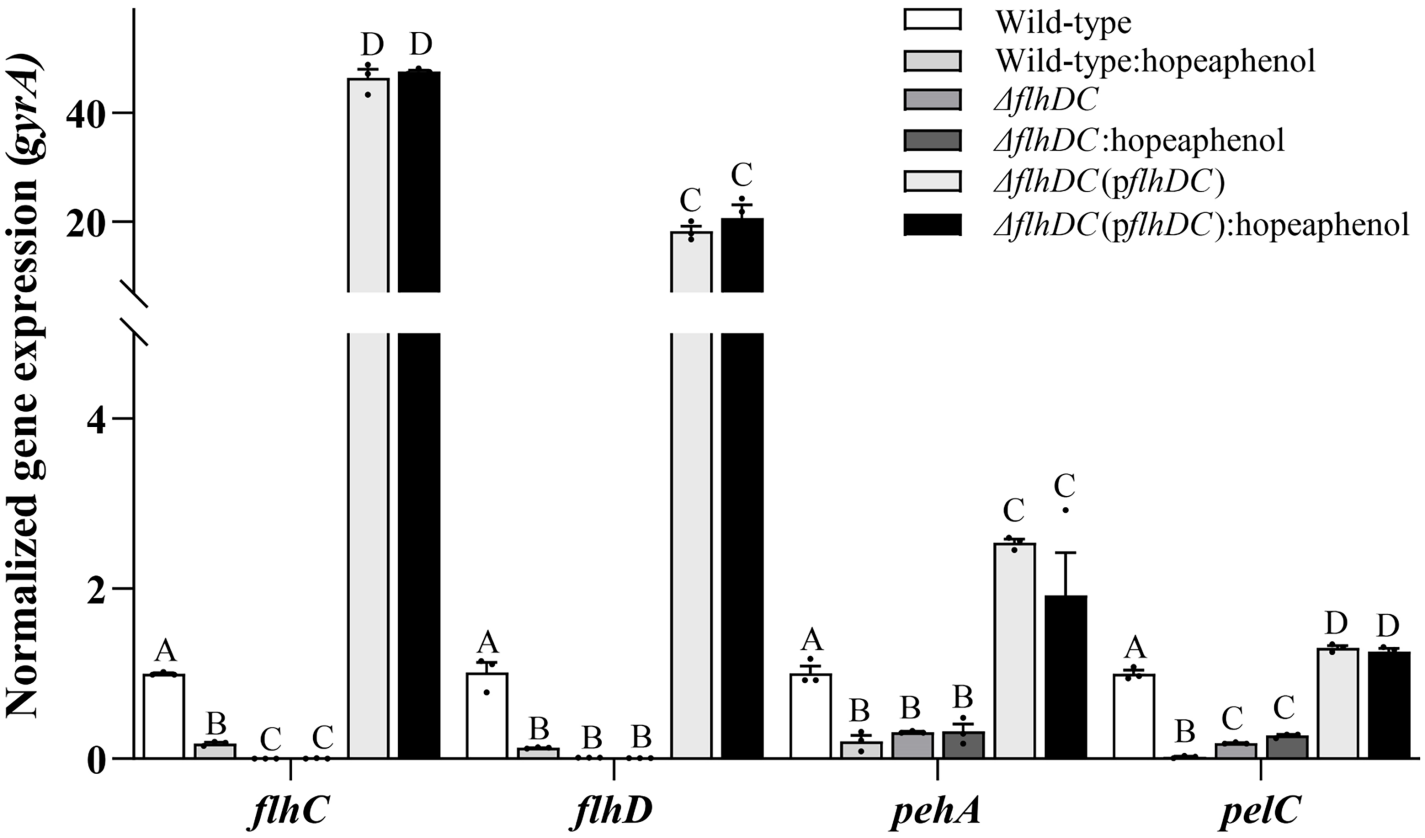
Transcriptional change of key virulence genes for a master regulator and extracellular enzymes in hopeaphenol-treated wild-type and *flhDC* mutant strain. Transcript levels of *flhDC, pehA* and *pelC* of *Pba* strains (wild-type, Δ*flhDC*, Δ*flhDC*(p*flhDC*)) with or without hopeaphenol were analyzed by qRT-PCR. The expression of each gene was normalized to the internal control gene, *gyrA*. The data represented three independent experiments with the mean of relative expression. Error bars demonstrated the standard deviation of three replicates. Different letters on the graph indicate statistical difference analyzed by one-way ANOVA with LSD test (*p* < 0.05).

The expression of *Pba* genes responsible for extracellular enzyme production, *pelC* (encoding pectate lyase), *pehA* (encoding polygalacturonase) and the master regulator genes (*flhC* and *flhD),* was analyzed by qRT-PCR. Wild-type strain expressed less *flhC, flhD, pelC* and *pehA* genes in the presence of hopeaphenol (Figure 4). This observation was consistent with an inhibitory effect of hopeaphenol on extracellular enzyme secretion of wild-type strain that hopeaphenol suppressed secretion of Pel and Peh as shown in Table 1. In Δ*flhDC* strain, the expression of *pelC* and *pehA* decreased compared to wild-type strain, but inhibitory effect of hopeaphenol on their expression in Δ*flhDC* strain was not observed due to very low expression (Figure 4). The complemented strain demonstrated much higher transcriptional levels of *flhC, flhD, pelC* and *pehA* than the gene expression level in wild-type strain (Figure 4). Together, these results revealed that hopeaphenol has an inhibitory effect on the secretion of exoenzymes in *Pba* and FlhDC involved in the exoenzyme secretion for the virulence activity with the inhibitory effect on the expression of *flhC, flhD, pelC* and *pehA.*

### Hopeaphenol suppresses *Pba*-triggered potato blackleg disease

**Figure 5:**
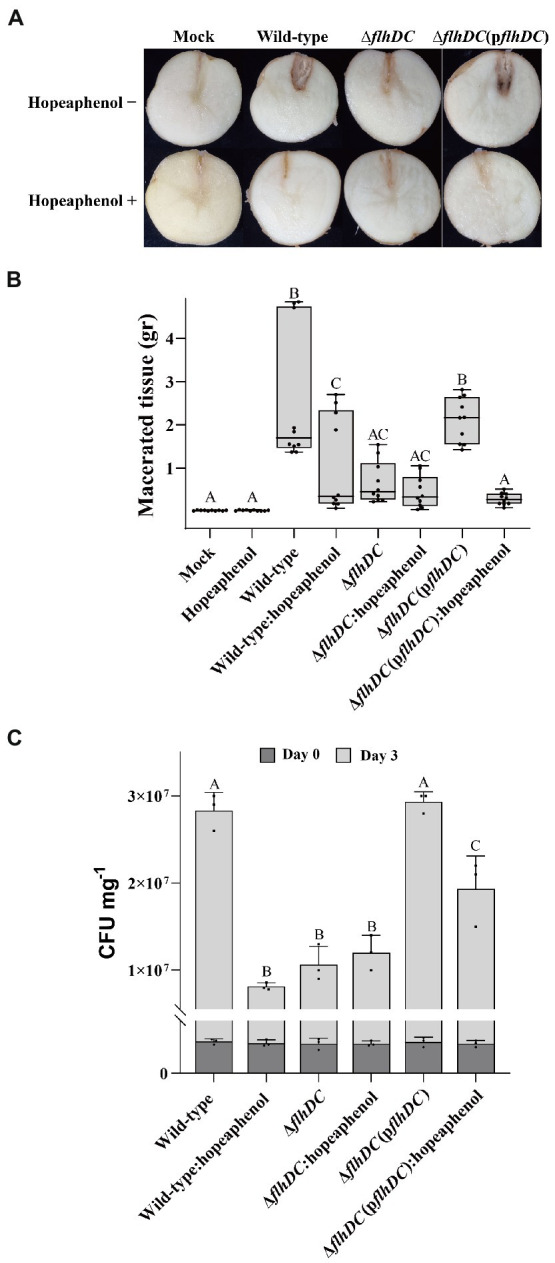
*Pba*-inducing disease symptoms are suppressed by hopeaphenol-treatment and in Δ*flhDC* strain. **(A)** Blackleg symptoms in potato tubers infected by *Pba* strains (wild-type, Δ*flhDC*, Δ*flhDC*(p*flhDC*)) with or without hopeaphenol. The symptom was observed 3 days post-infection. Similar results were obtained in three independent experiments with five replicates. **(B)** Maceration levels of potato tubers challenged with *Pba* strains. Fresh weight (g) of the macerated region in potato tuber was measured 3 days post infiltration with 10 mM of MgCl_2_ (mock) or bacterial suspension with or without 100 μM of hopeaphenol. Box plots present three independent experiments (n = 10) with statistical significances analyzed by One-way ANOVA with LSD test (*p* < 0.05). **(C)** Quantification of *Pba* growth in the macerated potato tuber. The bacterial population in potato tuber inoculated with each *Pba* strain was determined 0 and 3 days after infiltration. Error bars indicate the standard deviation (SD) of three replicates. Statistical analysis was performed by One-way ANOVA (*p* < 0.05).

Based on the earlier results that hopeaphenol dampened *Pba* motility and extracellular enzyme secretion (Figure 3 and Table 1), we expected that hopeaphenol could affect *Pba*-triggering potato blackleg disease symptoms by modulating the role of master regulator function and suppressing *Pba* virulence functions such as cell motility and extracellular enzyme activity. To confirm this hypothesis, we examined the effect of hopeaphenol on potato blackleg disease induced by *Pba*. Bacterial strains used in the previous experiments including wild-type, double mutant, and complemented strain were inoculated into potato tubers with or without hopeaphenol (Figure 5). Blackleg symptom was clearly reduced in mutant *Pba* strain (Δ*flhDC*), whereas the complemented stain (Δ*flhDC*(p*flhDC*) caused similar disease symptom as in wild-type strain. As expected, the disease symptom was decreased in potato tubers inoculated by all *Pba* strains upon hopeaphenol-treatment (Figure 5A, bottom). In Figure 5B, the macerated tissue in potato tuber inoculated with Δ*flhDC* or hopeaphenol-treated wild-type strain was suppressed dramatically, as similarly observed in the visual symptoms represented in Figure 5A. Lastly, we directly quantify the bacterial population infected by each *Pba* strains (Figure 5C). The bacterial population 3 days post inoculation with wild-type and complemented strain was greater than Δ*flhDC* mutant. With hopeaphenol-treatment, the bacterial population of wild-type and complementation strain in potato tubers reduced significantly. Hence, these results strongly suggest that hopeaphenol can suppress disease development in *Pba-*inoculated potato tubers by interrupting the function of the master regulator FlhDC required for the development of potato blackleg disease.

## Discussion

Plant-derived stilbene compounds possess diverse biological activities beneficial for human health and plant protection from environmental stress. Stilbene compounds target bacterial virulence as putative replacement or supplement for the use of conventional antibiotics that often induce pathogen resistance (Silva et al., 2016). The stilbenes also target quorum sensing, biofilm formation, motility and secretion systems in bacteria (Silva et al., 2016). Previous work reported that some of the resveratrol derivatives including resveratrol, piceatannol, rhaponticin, pallidol, alopecurone, ampelopsin A, kobophenol A, hopeaphenol, and isohopeaphenol inhibit promoter activity of *hrp* pilus gene in *Pst* DC3000 that implies potential T3SS inhibitory activity of stilbene compounds (Kang et al., 2020). By employing one of stilbene compounds, hopeaphenol, we further investigated anti-virulence activity against *Pba* SCRI1043 observing that hopeaphenol repressed bacterial motility and secretion of extracellular enzymes without growth retardation. Similarly to our results, the activity of stilbenes on bacterial motility was demonstrated in human bacterial pathogens (Wang et al., 2006; Sheng et al., 2015; Bostanghadiri et al., 2017). Resveratrol suppresses several motility and flagellar genes such as *flhD, fimA, fimH* and *motB* of *E. coli* O157:H7 (EHEC) and swarming motility of *P. aeruginosa* PAO1 (Sheng et al., 2015; Bostanghadiri et al., 2017). In *Proteus mirabilis* infecting urinary tract, resveratrol acts on a two-component system possibly involved in bacterial quorum-sensing mediating swarming and expression of virulence factors (Wang et al., 2006). However, application of stilbene compounds as anti-virulence agents to manage plant diseases has not been intensively studied yet. Thus, our study proposes a potential application of stilbene compounds for crop management and plant health as an alternative of antibiotics.

Hopeaphenol was previously isolated from *Vitis vinifera* to have an inhibitory effect on T3SS of a hemi-biotrophic pathogen, *Pst* DC3000 and disease development in tomato plants (Kang et al., 2020). Expression of three T3SS-related genes including *hrpA* (encoding *hrp* pilus), *hrpL* (encoding alternative sigma factor) and *hopP1* (encoding lytic transglycosylase) in *Pst* DC3000 were down-regulated by hopeaphenol, protecting tomato plants from *Pst* DC3000 (Kang et al., 2020). Based on this, it is highly possible that hopeaphenol can inhibit T3SS of *Pectobacterium* that possesses T3SS. However, the transcriptomic analysis uncovered that hopeaphenol triggered transcriptional repression of key virulence genes involved in flagellar motility and bacterial type II (T2SS) and type VI secretion systems (T6SS) (Supplementary Data sheet 2). The T6SS has been studied in some plant and animal pathogens for its role in bacteria competitions (Poole et al., 2011; Russell et al., 2014). *P. atrosepticum* activates the T6SS in presence of potato tuber extract (Mattinen et al., 2007). Type VI effectors of *P. carotovorum* subsp. *brasiliense* inhibit growth of some bacteria including *D. chrysanthemi, D. dadantii,* and *P. carotovorum* subsp. *carotovorum* (Shyntum et al., 2019). However, it is still elusive how bacterial T6SS and effectors can modulate the virulence of plant bacterial pathogens in the host plants. Our transcriptomic results can provide a potential research interest to further investigate the relation between T6SS and pathogen virulence activity and the role of hopeaphenol in T6SS.

The master regulator operon *flhDC* is required for biogenesis of cell surface flagella for bacterial motility and the production of plant cell wall-degrading enzymes (PCWDEs) secreted mainly by type I and T2SS (Reverchon et al., 2016). FlhDC activates the expression of the genes for motility such as *fliA* and *rsmB* RNA controlling PCWDEs production, and key regulatory (*gacA*) and sigma factor genes (*hrpL*) associated with virulence of *P. carotovorum* (Cui et al., 2008). Consistent with this, mutant in *flhDC* genes of *Pba* SCRI1043 constructed by CRISPR-Cas9 technique newly employed in this study demonstrated impaired motility and reduced extracellular enzyme production as a phenocopy of hopeaphenol-treated wild-type strain. However, transcription and secretion of extracellular enzymes such as pectate lyase, PelC and polygalacturonase, PehA were much more affected by hopeaphenol than deletion of *flhDC* genes. Thus, we infer that the regulation of PCWDEs in *Pba* SCRI1043 can be controlled by either *flhDC-*dependent and *-*independent pathway, and the effect of hopeaphenol on the production of PCWDEs can affect both pathways. In addition, our transcriptomic analysis represented that hopeaphenol modulated the transcription of several genes for T2SS through which PCWDEs can export from bacterial cell to host plants (Islam et al., 2019). This suggests that hopeaphenol might regulate the transcription and secretion of extracellular enzymes. In the complemented *Pba* strain, we noted that the highly overexpressed *flhDC* cannot fully suppressed by hopeaphenol as observed in wild-type strain. However, hopeaphenol retained the ability to suppress extracellular enzyme-dependent virulence activity, leading us to speculate that hopeaphenol may affect enzymatic secretion and activity. However, further study will be necessary to prove this. Disease severity of blackleg decreased in potato tuber infected by *Pba* strains with hopeaphenol, proposing that the reduction in the enzyme production by treatment of hopeaphenol or deletion of *flhDC* genes resulted in attenuation of blackleg disease in potato tuber. Therefore, we infer that hopeaphenol can modulate production of extracellular enzymes and the master regulator directly or indirectly, leading to attenuation of disease symptoms in potato tubers.

In sum, FlhDC is necessary for the movement of *Pba* and sufficient for extracellular enzyme production. Hopeaphenol regulates both bacterial movement and extracellular enzyme production/secretion, attenuating disease symptoms and development in potato tubers. Our findings delineate the role of FlhDC in *Pba* and the potential of natural stilbene as a source for bacterial disease management, bypassing the use of antibiotics. We also prospect further studies to understand the fine-tuned mechanism of FlhDC in *Pba* and the role of hopeaphenol on bacterial secretion system in terms of pathogen virulence.

## Data availability statement

The datasets presented in this study can be found in online repositories. The names of the repository/repositories and accession number(s) can be found below: NCBI Gene Expression Omnibus accession number: GSE196675.

## Author contributions

JK, SH, and NY performed the experiments. JK, SH, NY, and E-HC analyzed the results. JK, SH, and E-HC conceptualized and wrote the draft. JK, SH, BK, and E-HC edited the final manuscript. All authors contributed to the article and approved the submitted version.

## Funding

This work was supported by the BK21 FOUR program (Grant No. 4299991014324). JK was funded by the National Research Foundation of Korea (NRF-2021R1A6A3A01086358). E-HC was supported by the Korea University Grant (K2021521 and K2106871), the Korea University Insung Research Grant (K2128041), and Rural Development Agent (PJ015871032021).

## Conflict of interest

The authors declare that the research was conducted in the absence of any commercial or financial relationships that could be construed as a potential conflict of interest.

## Publisher’s note

All claims expressed in this article are solely those of the authors and do not necessarily represent those of their affiliated organizations, or those of the publisher, the editors and the reviewers. Any product that may be evaluated in this article, or claim that may be made by its manufacturer, is not guaranteed or endorsed by the publisher.

## Footnotes

^1^ https://www.bioinformatics.babraham.ac.uk/projects/fastqc

^2^ https://www.ncbi.nlm.nih.gov/genome/1088?genome_assembly_id=300523
