# Supplementary material for: A resveratrol oligomer, hopeaphenol suppresses virulence activity of Pectobacterium atrosepticum via the modulation of the master regulator, FlhDC
Source: Front Microbiol. 2022 Oct 28;13:999522. doi: 10.3389/fmicb.2022.999522 (PMC9650432; doi:10.3389/fmicb.2022.999522)
Supplement: Supplementary file 1 [file Data_Sheet_1.DOCX]

**Supplementary Figure 1**. Transcription of a master regulator (*flhC* and *flhD* genes) supplemented with 100 μM of hopeaphenol in *Pba* SCRI1043. The transcriptional level of a housekeeping gene, *gyrA* was used as an internal control. The bar graph represents the means of transcriptional levels of each gene with standard deviation (SD). One-way ANOVA was used for statistical analysis and asterisks indicate statistic differences (*P*<0.05).


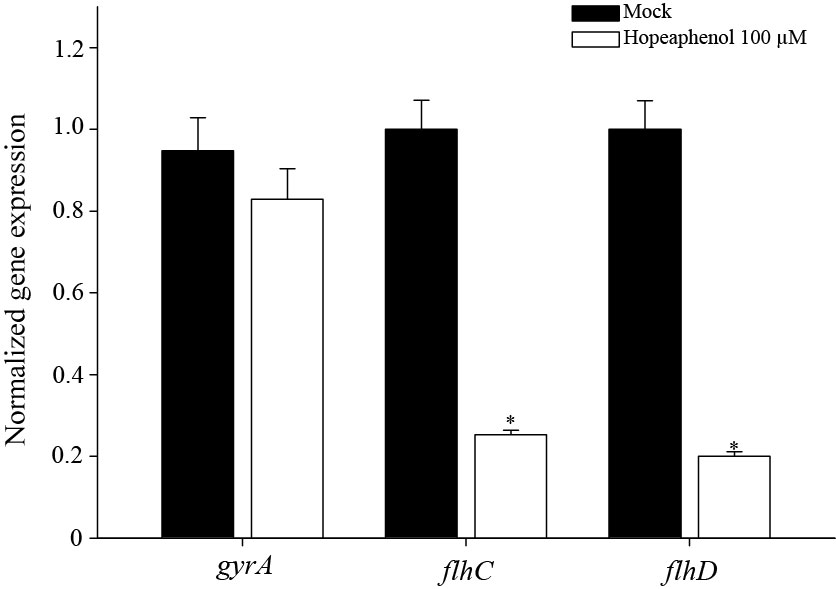


**Supplementary Figure 2.** Transcription of a master regulator (*flhC* and *flhD* genes) in *Pba* strains (Wild-type, Δ*flhDC*, Δ*flhDC*(p*flhDC*)). The transcriptional level of a housekeeping gene, *gyrA* was used as an internal control. The bar graph represents the means of transcriptional levels of each gene with standard deviation (SD).


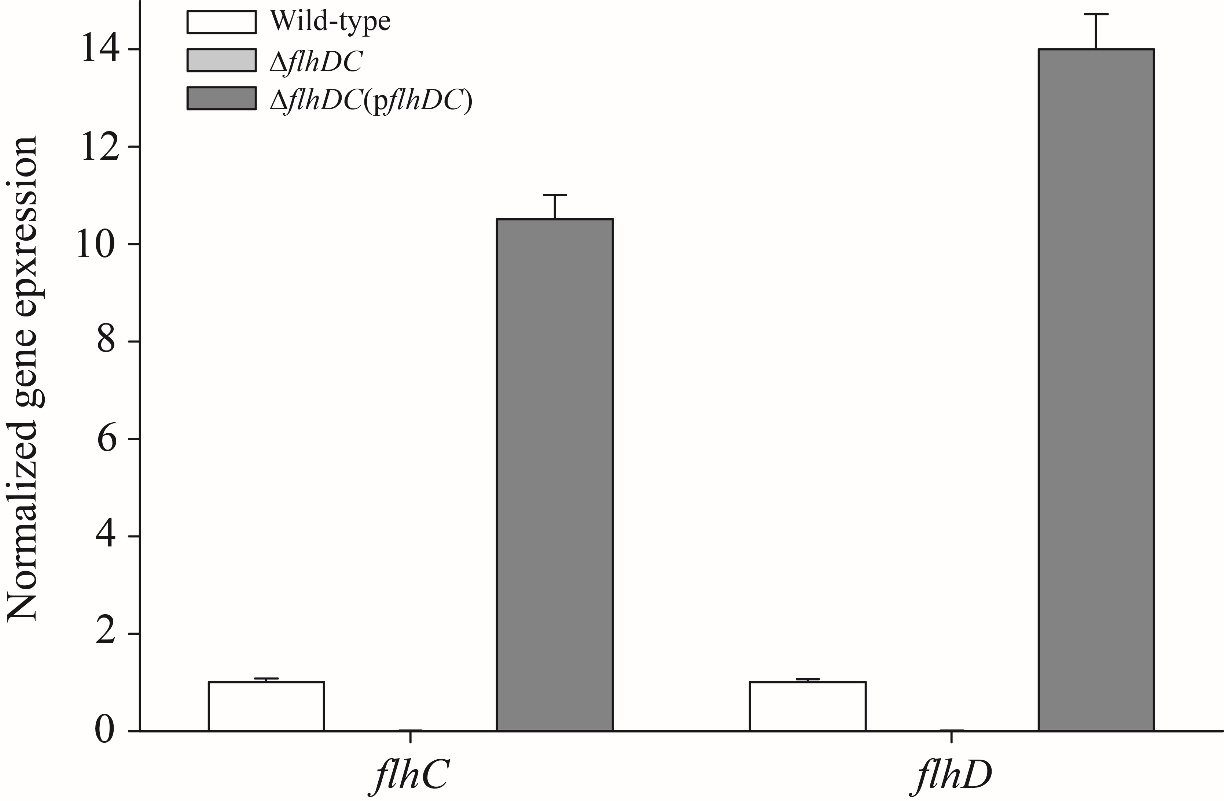


**Supplementary Table 1.** Oligonucleotides and primers used in this study.

| **Oligonucleotide** | **Sequences** | |
| --- | --- | --- |
| Spacer | TAGTTACAATCAAAGTGTTGACCC | |
| ssDNA | CCGCAGATGGTTAAATTGGCAGAAACTAACCAACTGATATGTTCAGGAAGCGAAAGACATCCAATTGGCGATGGAGCTCA | |
|  | | |
| **Gene** | **Forward (5’-3’)** | **Reverse (5’-3’)** |
| *flhDC* | CTTTGTACGCATTTCTGACG | TGTTGTCGGAAAGGATATTCG |
| *pelC* | CGCTGGGCTGTTATTACTTG | TCGATAATATCAATGATATCTTGCATAG |
| *pehA* | CATTATCATTGGGCCTTATCG | CGCAATTATTCAGTGCTTTTTG |
| *flhC* | GGCATAAGTAATGGCGGAGA | AAACGTCCGCGACTTAACTG |
| *flhD* | AAGCTTCTGCGATGTTTCGT | ATCCCCGTATGAATTTGCTG |
| *gyrA* | GTTGATGGTCAGGGCAACTT | TTAACCAGCAGGTTGGGAAC |

**Supplementary Table 3.** Functional enrichment analysis of differentially expressed genes by hopeaphenol in *Pba* SCRI1043

| **DEGs** | **COG categories** | **COG.padj** | **Number** |  |
| --- | --- | --- | --- | --- |
| All | CK | 0 | 1 | **INFORMATION STORAGE AND PROCESSING** **[A]** RNA processing and modification **[B]** Chromatin structure and dynamics  **[J]** Translation, ribosomal structure and biogenesis **[K]** Transcription **[L]** Replication, recombination and repair  **CELLULAR PROCESSES AND SIGNALING** **[D]** Cell cycle control, cell division, chromosome partitioning **[Y]** Nuclear structure **[V]** Defense mechanisms **[T]** Signal transduction mechanisms **[M]** Cell wall/membrane/envelope biogenesis **[N]** Cell motility **[Z]** Cytoskeleton **[W]** Extracellular structures **[U]** Intracellular trafficking, secretion, and vesicular transport **[O]** Posttranslational modification, protein turnover, chaperones  **METABOLISM** **[C]** Energy production and conversion **[E]** Amino acid transport and metabolism **[F]** Nucleotide transport and metabolism **[G]** Carbohydrate transport and metabolism **[H]** Coenzyme transport and metabolism **[I]** Lipid transport and metabolism **[P]** Inorganic ion transport and metabolism **[Q]** Secondary metabolites biosynthesis, transport and catabolism  **POORLY CHARACTERIZED** **[R]** General function prediction only **[S]** Function unknown |
| All | TU | 0 | 1 |  |
| All | EP | 0 | 2 |  |
| All | NPTU | 0 | 2 |  |
| All | NT | 1.55E-10 | 23 |  |
| All | N | 7.48E-10 | 24 |  |
| All | G | 3.08E-07 | 63 |  |
| Down | EJ | 0.039 | 1 |  |
| Down | NU | 0.042065 | 6 |  |
| Down | NT | 2.42E-13 | 20 |  |
| Down | U | 6.11E-05 | 21 |  |
| Down | N | 2.22E-16 | 24 |  |
| Up | CK | 0 | 1 |  |
| Up | EP | 0 | 2 |  |
| Up | NPTU | 0 | 2 |  |
| Up | G | 1.46E-08 | 42 |  |

**Supplementary Table 4.** Up-regulated genes clustered into 13 metabolic pathways by the pathway enrichment.

| **Pathway** | **hit.ratio** | **hits** | **selected** | **hit.big** | **background** | ***p* value** | ***q* value** |
| --- | --- | --- | --- | --- | --- | --- | --- |
| eca00071 Fatty acid degradation | 0.636363636 | 7 | 118 | 11 | 1632 | 2.25E^-06^ | 0.000254442 |
| eca00281 Geraniol degradation | 0.8 | 4 | 118 | 5 | 1632 | 0.000122991 | 0.006825728 |
| eca00280 Valine, leucine and isoleucine degradation | 0.555555556 | 5 | 118 | 9 | 1632 | 0.000181214 | 0.006825728 |
| eca00500 Starch and sucrose metabolism | 0.229166667 | 11 | 118 | 48 | 1632 | 0.000404356 | 0.011423047 |
| eca00410 β-Alanine metabolism | 0.571428571 | 4 | 118 | 7 | 1632 | 0.000767451 | 0.017344402 |
| eca00310 Lysine degradation | 0.416666667 | 5 | 118 | 12 | 1632 | 0.000953639 | 0.017960203 |
| eca01120 Microbial metabolism in diverse environments | 0.127272727 | 28 | 118 | 220 | 1632 | 0.001199313 | 0.019360338 |
| eca00650 Butanoate metabolism | 0.242424242 | 8 | 118 | 33 | 1632 | 0.001764196 | 0.02246766 |
| eca00620 Pyruvate metabolism | 0.204081633 | 10 | 118 | 49 | 1632 | 0.001957603 | 0.02246766 |
| eca02020 Two-component system | 0.132947977 | 23 | 118 | 173 | 1632 | 0.001988288 | 0.02246766 |
| eca00040 Pentose and glucuronate interconversions | 0.228571429 | 8 | 118 | 35 | 1632 | 0.002644662 | 0.027167892 |
| eca00362 Benzoate degradation | 0.4 | 4 | 118 | 10 | 1632 | 0.003878982 | 0.036527078 |

**Supplementary Table 5.** Down-regulated genes clustered into 6 categories by the pathway enrichment.

| **Pathway** | **hit.ratio** | **hits** | **selected** | **hit.big** | **background** | ***p* value** | ***q* value** |
| --- | --- | --- | --- | --- | --- | --- | --- |
| eca02040 Flagellar assembly | 0.555555556 | 25 | 152 | 45 | 1632 | 1.63E^-15^ | 1.84E^-13^ |
| eca02030 Bacterial chemotaxis | 0.5 | 24 | 152 | 48 | 1632 | 1.50E^-13^ | 8.46E^-12^ |
| eca03070 Bacterial secretion system | 0.338709677 | 21 | 152 | 62 | 1632 | 3.92E^-08^ | 1.48E^-06^ |
| eca02020 Two-component system | 0.167630058 | 29 | 152 | 173 | 1632 | 0.000710503 | 0.018486891 |
| eca00020 Citrate cycle (TCA cycle) | 0.310344828 | 9 | 152 | 29 | 1632 | 0.000818004 | 0.018486891 |
| eca00730 Thiamine metabolism | 0.375 | 6 | 152 | 16 | 1632 | 0.002153614 | 0.040559734 |
